# Supplementary material for: Effects of multimodal explanations for autonomous driving on driving performance, cognitive load, expertise, confidence, and trust
Source: Sci Rep. 2024 Jun 6;14:13061. doi: 10.1038/s41598-024-62052-9 (PMC11156640; doi:10.1038/s41598-024-62052-9)
Supplement: Supplementary file 1 — Supplementary Information. [file 41598_2024_62052_MOESM1_ESM.pdf]

Supplementary Information

Measures

Trust in AV Driving Coach (Strongly disagree – Strongly agree, 5-point)
I like the idea of an AI driving coach.
I would trust the advice given to me by an AI driving coach.
An AI driving coach can help me become a better driver.
AI can be a useful learning tool.

General Trust in AVs (Strongly disagree – Strongly agree, 5-point)
Overall, I feel positive about autonomous vehicles.
I would trust an autonomous vehicle to drive me around safely.
I know when I can rely on autonomous vehicles and when I cannot.
I understand why an autonomous vehicle makes decisions.

Self-Reported Performance Driving Expertise (Strongly disagree – Strongly agree, 5-point)
I understand the concepts behind performance driving.
I am an expert at performance driving.
I am interested in learning to be a better performance driver.

Racing Line Knowledge (True/False/Unsure)
You generally want to take the straightest driving path you can while going around the track. T
It is generally optimal to be in the center of the track as opposed to on the edge. F
It is generally best to enter a curve to the left from the right side of the track. T
In performance driving, it is unacceptable to touch the tires to the edge of the track. F
Which colored line is the best path around this corner?(Yellow, Red, Green) Red*
**This question displayed a road turning to the right with three colored lines overlayed on it: a yellow line following the left outside curb, a green line following the right inside curb, and a red line that followed from outside by yellow before the turn, to inside by green at the turn apex, and back outside again after the turn finished.*

Performance Driving Confidence (0-10 scale)
If you were asked to performance drive in real life (without assistance), how confident would you feel?

Semi-Structured Interview Script Example

- *What did you think of your experience?*
  - *Probe for: realism and immersion*
- *Can you take me through your learning process?*
  - *How did the AI coach help or hurt this process?*
- *In terms of learning to get better over time–*
  - *Did you feel like you were improving?*
  - *What was helping you improve or keep you from improving?*
- *Can you tell me your thoughts about the AI coach observation sessions?*
  - *Were they helpful for you? Why or why not?*
  - *What was helpful and not?*
  - *Is there any other information that would have been helpful for you to learn better?*
- *What are your thoughts and impressions about AI vehicles in general? AI coaches in general?*
  - *Probe for trust, etc.*
